# Cold Atmospheric Plasma and Silymarin Nanoemulsion Activate Autophagy in Human Melanoma Cells

**DOI:** 10.3390/ijms21061939

**Published:** 2020-03-12

**Authors:** Manish Adhikari, Bhawana Adhikari, Bhagirath Ghimire, Sanjula Baboota, Eun Ha Choi

**Affiliations:** 1 Plasma Bioscience Research Center, Applied Plasma Medicine Center, Department of Electrical and Biological Physics, Kwangwoon University, Seoul 01897, Korea; bnegi87@gmail.com (B.A.); ghimirebhagi@hotmail.com (B.G.); 2 Department of Pharmaceutics, School of Pharmaceutical Education and Research, Jamia Hamdard, Delhi 110062, India; sbaboota@rediffmail.com

**Keywords:** cold atmospheric plasma, autophagy, silymarin nanoemulsion, *PI3K/mTOR* pathway

## Abstract

Background: Autophagy is reported as a survival or death-promoting pathway that is highly debatable in different kinds of cancer. Here, we examined the co-effect of cold atmospheric plasma (CAP) and silymarin nanoemulsion (SN) treatment on G-361 human melanoma cells via autophagy induction. Methods: The temperature and pH of the media, along with the cell number, were evaluated. The intracellular glucose level and *PI3K/mTOR* and *EGFR* downstream pathways were assessed. Autophagy-related genes, related transcriptional factors, and autophagy induction were estimated using confocal microscopy, flow cytometry, and ELISA. Results: CAP treatment increased the temperature and pH of the media, while its combination with SN resulted in a decrease in intracellular ATP with the downregulation of *PI3K/AKT/mTOR* survival and *RAS/MEK* transcriptional pathways. Co-treatment blocked downstream paths of survival pathways and reduced *PI3K* (2 times), *mTOR* (10 times), *EGFR* (5 times), *HRAS* (5 times), and *MEK* (10 times). CAP and SN co-treated treatment modulates transcriptional factor expressions (*ZKSCAN3*, *TFEB*, *FOXO1*, *CRTC2*, and *CREBBP*) and specific genes (*BECN-1*, *AMBRA-1*, *MAP1LC3A,* and *SQSTM*) related to autophagy induction. Conclusion: CAP and SN together activate autophagy in G-361 cells by activating *PI3K/mTOR* and *EGFR* pathways, expressing autophagy-related transcription factors and genes.

## 1. Introduction

Malignant melanoma arises from the irregular dysfunction of human skin pigment-producing cells (melanocytes), which results in the overproduction of skin color [1]. Around half of melanoma carry a mutation in the *BRAF* gene, which results in the dysregulation of several molecular signaling pathways [2]. Till now, various signaling pathways involved in resistance, as well as the spectrum of therapy-acquired mutations, have been known, while the cellular aspects of therapy resistance have not [3]. Antecedently, remarkable progress was achieved in both immunotherapy and molecular-targeted therapy as the standard of care for terminal melanoma patients [4,5]. Cold atmospheric plasma (CAP) is an evolving biomedical technique that has been used in multifarious ways, like in skin disorders, wound healing, dentistry, hair treatment, various kinds of cancers, etc., in past years [6,7,8,9,10]. It is well-known that CAP generates reactive oxygen and nitrogen species (RONS), UV rays, and charged particles that physically and chemically change the biological surfaces by inducing oxidative stress, which results in a change in certain gene expression and epigenetic changes [11,12]. Our previous publication revealed the generation of RONS using a micro-dielectric barrier discharge (μ-DBD) device (same device used for this study), which aided in the reduction of melanoma cells by apoptosis [11]. In the last few years, nanotechnology has also played a key role in cancer treatment [13]. Our group recently published work demonstrating the synergistic effects of gold nanoparticles with CAP and silymarin nanoemulsion with CAP in which glioblastoma and human melanoma were killed by inhibiting the *PI3K/AKT* pathway and *HGF/c-MET* pathway, respectively [11,14]. Using a nanotechnology and pharmacology approach, we prepared, characterized, and performed experiments on silymarin nanoemulsion (SN), which was used in our previous publication [11]. The protein expression levels and associated signaling of the *PI3K/MEK* and *HRAS* pathways using CAP and SN have already been described and presented in our previous publication [11]. Silymarin is widely well-accepted as a heptoprotectant and reported in the treatment of different kinds of cancer [15,16]. SN has been shown to decrease silymarin hydrophobicity by its heterogeneous dispersion of two immiscible liquids (oil-in-water) and hence improve the bioavailability. In the past few years, nanotechnology combined with CAP technology has exhibited some promising aspects in cancer research by the activation/deactivation of cellular pathways [17]. Additionally, it has been proposed by some research groups that CAP can help in selective cell membrane permeability, which helps in the invasion of CAP-generated direct and indirect RONS species, leading to the intracellular invasion of nanoparticles towards applied sites [18,19,20]. However, at the clinical level, the outcome of the survival of melanoma patients has been very limited due to the targeted mutation in molecular targeted therapy [21]. Cell growth inhibition and cell death induction are the main objectives of every successful cancer treatment.

Melanoma seems to be a specific type of cancer that dysregulates apoptosis and hence evolves as a cancer that is resistant to programmed cell death [22]. The cellular death (apoptosis) of G-361 human melanoma by treatment with CAP and SN was evaluated using the annexin V-PI staining method [11]. Therefore, the induction of another form of cellular death is necessary and fundamental to conquering this resistance [23,24]. Recent studies on various types of cancers have demonstrated that in premature tumors, cancer cells can upregulate a related stress response process called autophagy [25]. Autophagy is a vibrant cellular self-digestion cum destruction process and in normal cells, occurs at constitutive stages to maintain internal cellular homeostasis [26,27]. Research has presented substantial outcomes showing that the autophagy process plays a vital role against various diseases, cancer, aging, and neurodegenerative disease [28]. Hence, the initiation of other death mechanisms, such as autophagy, provides a critical defensive approach to guarantee the elimination of potential cancer cells [29]. These data, for the first time, highlight that CAP, along with SN treatment, leads to the accumulation of autophagosomes, which is the signal of autophagy induction that occurs by inhibiting the classical autophagy specific *mTOR* survival pathway and *MEK* pathway, respectively. Our data also suggests an increase in autophagy-specific genes and their related transcriptional factors, which could be developed as a new strategy for melanoma treatment.

Therefore, the current study is focused on manipulating autophagy in melanoma cells by a μ-dielectric barrier discharge (μ-DBD) air CAP device using air as feeder gas with SN by evaluating autophagy influx, transcriptional factors, and the activation of autophagy-specific genes. Hence, these results present evidence of a plausible role of the air CAP with SN delivery in inhibiting melanoma progression by the induction of autophagy, which may improve clinical outcomes of patients in the future.

## 2. Results

### 2.1. Electrical and Optical Characteristics of the μ-DBD Plasma Instrument

**Figure 1:**
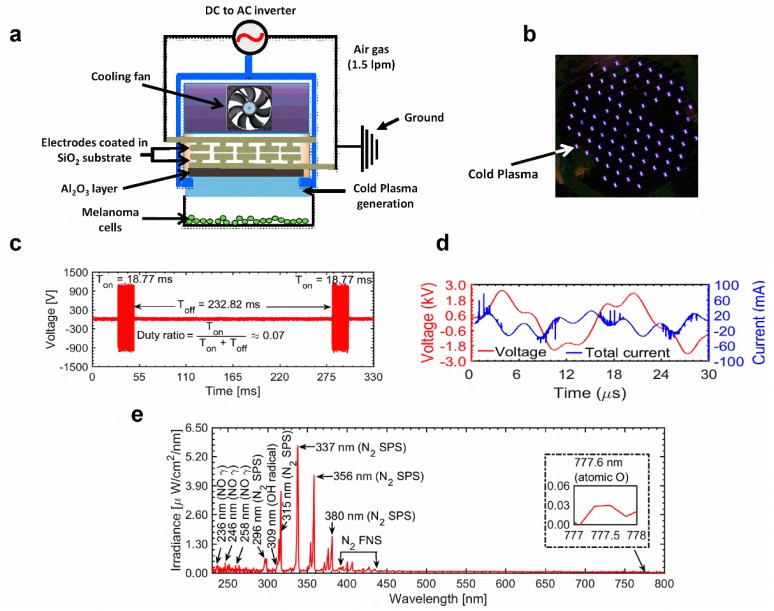
(**a**) Schematic diagram of the non-thermal cold atmospheric plasma (CAP) micro-dielectric barrier discharge (μ-DBD) plasma source with a distance of 2 mm from cell culture media. (**b**) Photograph of the μ-DBD plasma device showing plasma discharge using air as the feeder gas. (**c**) The μ-DBD device on time (T_on_) and off-time (T_off_). (**d**) Voltage and current waveforms for one cycle of the T_on_ period of the μ-DBD air CAP device. (**e**) Optical emission spectrum (OES) composition of the air μ-DBD CAP source between 200 nm and 800 nm.

The electrical and optical characteristics of discharge are shown in Figure 1c–e. These waveforms were recorded using a Lecroy wave surfer 434, 350 MHz oscilloscope with a Tektronix P6015A high-voltage probe and Tektronix P6022 current probe. The discharge was operated in dimming mode by using a DC-AC inverter whose operational time (T_on_) and shutting time (T_off_) were set to 18.77 ms and 232.82 ms, respectively. These waveforms are shown in Figure 1c. The duty percentage was ≈7% and the repetition frequency was ≈4 Hz. A longer shutting off time between consecutive discharges of short durations could enable longer operation of the source, without sufficient heating of the electrodes. The operational time of the discharge for the μ-DBD plasma source is characterized by repetitive sinusoidal waveforms that have a frequency of ≈60 kHz. These waveforms for voltage and current are shown in Figure 1d. Every positive half cycle of the applied voltage, a number of micro-discharges are initiated, which causes the positive polarity current. During this process, charges are accumulated on the surface of dielectric material and cause a negative polarity current during the negative half cycle. The values of the applied voltage (rms) and total current (rms) were found to be 1.40 kV and 20 mA, respectively. The dissipated power in one duty cycle was calculated by integrating the current and voltage waveforms over one cycle of the discharge period ($P=duty-ratio\times \frac{1}{T}\int_{0}^{T}IVdt$) [30] and was found to be 0.40 J/s. 

The optical emission spectra (OES) of the µ-DBD air discharge recorded in the wavelength range of 200–900 nm is shown in Figure 1e. This spectrum was measured with an HR4000 spectrometer (Ocean Optics Corporation, Orlando, FL, USA). The conversion to absolute units (µW/cm^2^/nm) was performed by using a deuterium halogen lamp (Ocean Optics Corporation, Model: DH-3P-BAL-CAL). In order to record the OES spectra of the inhomogeneous discharge, the integration time of the spectrometer was set to 500 ms, with an average of 4. In the OES, it can be observed that there are weak emission signals from the nitric oxide gamma band (NO γ) at 236, 246, and 258 nm, etc. [31]. These species originated from the collision of energetic electrons/metastable atoms with air molecules. A weak emission signal from the hydroxyl radical (OH) can be observed in the range of 306–309 nm. OH radicals are formed through the dissociation of water molecules which might be present in the feeding gas and ambient environment [30]. Strong emissions from the nitrogen second positive system (N_2_ SPS) can be observed at 296, 315, 337, 356, and 380 nm, etc. There are also emissions from the nitrogen first negative system (N_2_ FNS) in the range of 390–440 nm [32]. The origin of excited nitrogen species can be attributed to the dissociation of nitrogen molecules which may be present in the feeding gas, as well as the ambient environment. In addition to these, there is also emission from the atomic oxygen (O) at 777 nm.

### 2.2. Effect of CAP on Physical Parameters (Extracellular pH and Temperature)

**Figure 2:**
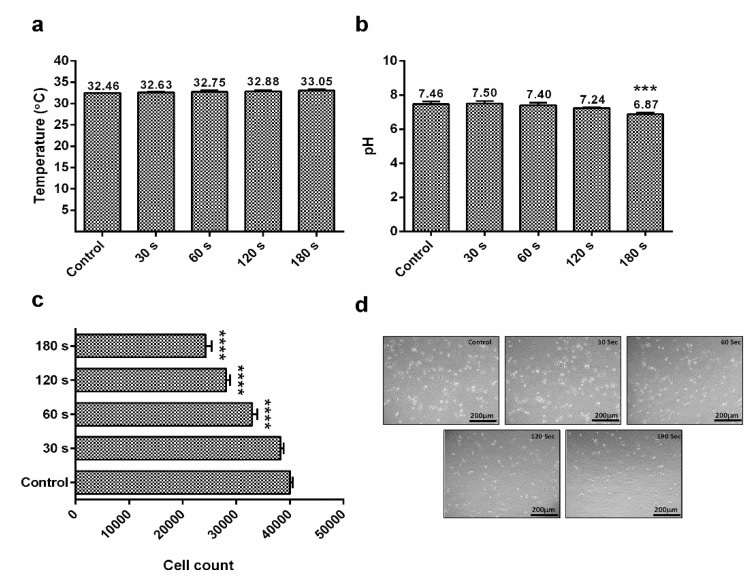
(**a**) Estimation of temperature within cell media (RPMI-1640) after the exposure of air CAP at different time intervals; (**b**) effect of different CAP doses on the pH level of cell media; (**c**) assessment of the G-361 human melanoma cell count using a hemocytometer counter after the exposure of CAP at different time intervals; (**d**) microscopic evaluation of G-361 human melanoma cells at different CAP doses (scale bar = 200 μm). Data are the mean ± SE of three experiments. Statistical analysis was performed using a one-way ANOVA test followed by Dunnett’s test for comparisons. Each asterisk represents statistical differences between the treatment and control (*** *p* < 0.001 and **** *p* < 0.0001).

Studies on different plasma devices revealed changes in the extracellular pH within cell culture media (RPMI) and its temperature. Here, the device (μ-DBD) shoots plasma in the form of dielectric barrier discharge which was prepared and characterized at the Plasma Bioscience Research Center, Seoul, South Korea. The temperature of RPMI-1640 cell culture media slightly increased when it received CAP for different time intervals. The CAP treatment time used for the extracellular pH and temperature estimation was 30, 60, 120, and 180 s, respectively. Initially, the recorded temperature was 32.63 °C (30 s), but it increased gradually up to 32.75 °C when treated with CAP for 60 s. Furthermore, treatment increased the extracellular temperature from 32.88 °C (120 s) to 33.05 °C (180 s) (Figure 2a).

With ambient air as the feeder gas for CAP generation, the extracellular pH (cell culture media) was 7.5 for 30 s, which was decreased by every plasma dose setup. The pH level was 7.40 (60 s), 7.24 (120 s), and 6.87 (180 s), respectively (Figure 2b). To check the reduction in G-361 cells, the CAP was treated at different time intervals (Figure 2c) and the cell count was recorded 24 h after the treatment. The cell count in control samples was 40,000, which kept on reducing while receiving CAP treatment and decreased to the minimum at 180 s treatment (24,300 counts), which was also evident from microscopy images of different CAP-treated groups. CAP treatment progressively killed the G-361 cells as the dose increased, which resulted in detachment and a round structure, with a reduction of cells at higher CAP doses (Figure 2d).

### 2.3. Intracellular ATP and Glucose Estimation

**Figure 3:**
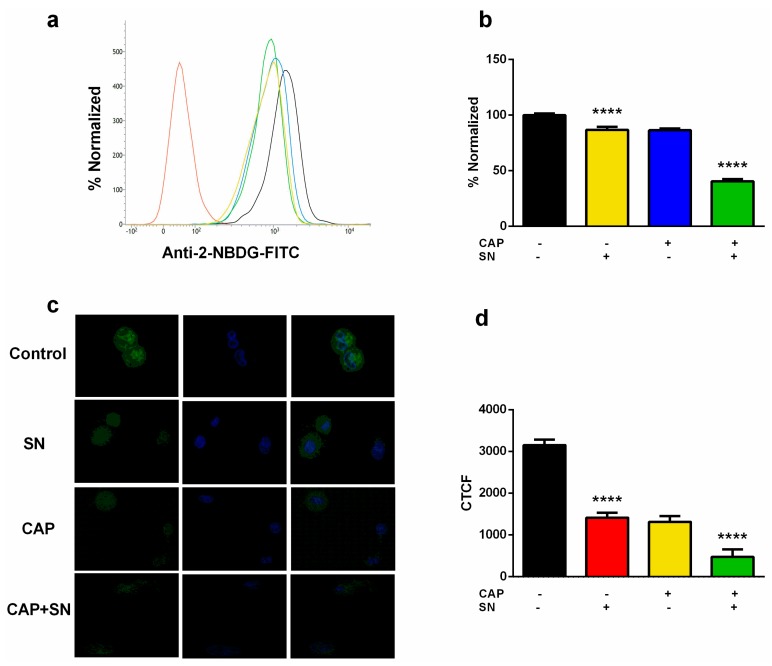
Estimation of intracellular glucose (ATP level) using fluorescent glucose analog 2-NBDG (**a**) employing flow cytometry in a bar graph; (**b**) calculated values in terms of the bar graph; (**c**) images were taken using confocal microscopy (intensity of green color represents the presence of glucose and blue indicates the nucleus); (**d**) captured images were expressed terms of Corrected Total Cell Fluorescence (CTCF) in a bar graph form. Data are the mean ± SE of three experiments. Statistical analysis was performed using a one-way ANOVA test followed by Dunnett’s test for comparisons. Each asterisk represents statistical differences between the treatment and control (**** *p* < 0.0001).

CAP induces an extracellular acidic environment which may lead to acidic stress within G-361 cells. This will propagate to intracellular metabolic stress and a limited nutrient supply. Cancer cells need more energy (ATP) and hence produce high glucose channels on the cell surface, which helps them to activate aerobic glycolysis for their proper functioning, as compared to normal adjacent healthy cells. Therefore, we assessed glucose uptake in G-361 melanoma cells after exposure to SN, CAP, and CAP + SN. We used 2-NBDG (2-deoxy-2-[(7-nitro-2,1,3-benzoxadiazol-4-yl)amino]-d-glucose), which is a fluorescent non-metabolizable glucose analog, to measure glucose uptake within melanoma cells. The flow cytometry histogram analysis-calculated bar graph showed that the level of glucose in SN and CAP slightly decreased (~80%) compared to the control. Additionally, the combination of CAP + SN drastically significantly reduced the glucose uptake level (~35%) (Figure 3a,b). 

The glucose uptake was also represented by confocal microscopy imaging, where the expression of glucose was shown in green and the nucleus was counterstained with blue (4′,6-diamidino-2-phenylindole (DAPI) stained). The signal of glucose (Green) was recorded and the Corrected Total Cell Fluorescence (CTCF) was recorded using Image J software (Figure 3c). The imaging data showed a significant reduction of glucose uptake in SN and CAP samples compared to the control. The combination group-calculated fluorescence value further declined significantly, which proves that a CAP and SN combination can decrease the glucose uptake level in melanoma (Figure 3d). 

### 2.4. PI3K Lead mTOR and EGF Signaling Arrest

*mTOR* is an important gene with a key role in the regulation of metabolism and cell growth. It is present in two different complexes: *mTORC1* (sensitive to rapamycin) and *mTORC2* (phosphorylates AKT and less sensitive to rapamycin). It also acts as a sensor for the availability of nutrients and glucose [33].

**Figure 4:**
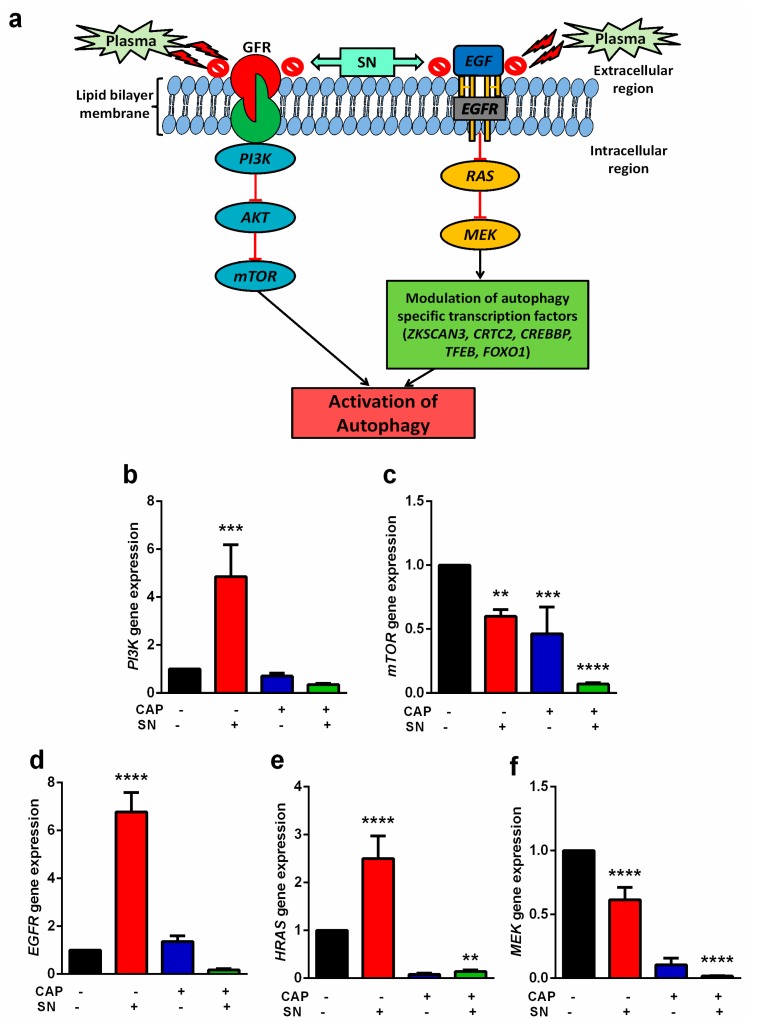
(**a**) Illustration of the CAP and silymarin nanoemulsion (SN)-mediated hypothesis about the blockage of *PI3K/AKT/mTOR* and *RAS/MEK* pathways for the activation of autophagy. Gene analysis of the *GFR*-mediated downstream expression of the (**b**) *PI3K* gene and (**c**) *mTOR* gene, and gene analysis of the Epidermal Growth factor (*EGF*)-mediated downstream expression of the (**d**) *EGFR* gene, (**e**) *HRAS* gene, and (**f**) *MEK* gene. Data are the mean ± SE of three experiments. Statistical analysis was performed using a one-way ANOVA test followed by Dunnett’s test for comparisons. Each asterisk represents statistical differences between the treatment and control (** *p* < 0.01, *** *p* < 0.001, and **** *p* < 0.0001).

According to our proposed hypothesis, CAP and SN co-treatment can block the *GFR* and *EGF* receptor, which eventually results in autophagy activation (Figure 4a). In normal conditions, *GFR* induces the classical *PI3K* pathway and leads to *mTOR*-mediated cell survival by inhibiting apoptosis. Another key receptor is the Epidermal Growth factor (EGF) receptor, which is known to be required for cell survival. *EGF* induces the expression of transcriptional factors, which further modulates autophagy.

To evaluate the molecular signaling events involved in the *mTOR* pathway, we analyzed the upstream events that regulate the *mTOR* pathway in melanoma cells exposed to CAP, SN, and CAP and SN co-treatment conditions. Our findings suggest that autophagy was well-activated by the blocking of these two classical pathways when co-treated with CAP and SN. We analyzed the lesser expression of downstream genes of both pathways when treated with CAP and SN. However, the SN-treated samples increased the level of all the *mTOR* pathway signaling genes, which means that SN treatment helps in autophagy regression and hence promotes melanoma progression. However, the level of *MEK* and *mTOR* in the SN-treated group showed a lower expression compared to the control. The level of the *PI3K* gene was significantly increased (5 times) in the SN-treated group compared to all other groups. CAP treatment reduced the level of *MEK* (0.1 times), *PI3K* (0.6 times), and *mTOR* (0.4 times) compared to the control (Figure 4b,c). However, CAP and SN treatment further decreased the expression levels of all mentioned genes, which led to the activation of autophagy. Other genes responsible for the activation of autophagy-specific transcriptional factors *EGFR* and *HRAS* were also increased significantly when they received SN treatment (Figure 4d–f). However, both genes were reduced significantly when co-treated with CAP and SN. The gene expression for *HRAS* was 0.2 fold and for *EGFR* was 0.3 fold, respectively, for the CAP and SN co-treated group compared to the control, suggesting that the activation of transcriptional factors is responsible for autophagy (Figure 4e,f).

### 2.5. Increase in Autophagic-Related Gene Expressions and Related Transcriptional Factors

**Figure 5:**
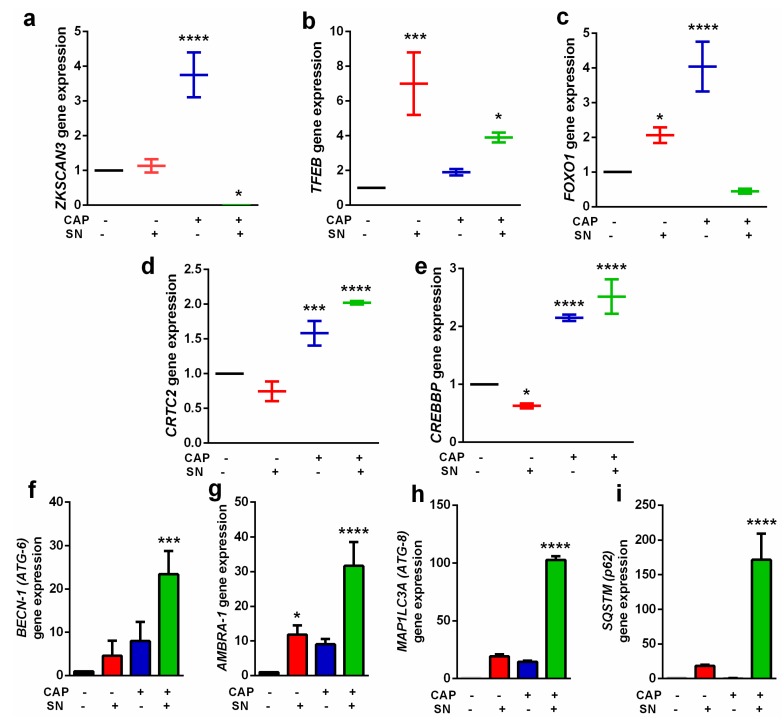
Gene expression levels of autophagy-related transcriptional factors: (**a**) *ZKSCAN3* gene; (**b**) *TFEB* gene; (**c**) *FOXO1* gene; (**d**) *CRTC2* gene; and (**e**) *CREBBP* gene. Expression of various gene levels directly responsible for autophagy: (**f**) *BECN-1 (ATG-6)*; (**g**) *AMBRA-1*; (**h**) *MAP1LC3A (ATG-8)*; and (**i**) *SQSTM (p62)*. Data are the mean ± SE of three experiments. Statistical analysis was performed using a one-way ANOVA test followed by Dunnett’s test for comparisons. Each asterisk represents statistical differences between the treatment and control (* *p* < 0.05, *** *p* < 0.001, and **** *p* < 0.0001).

In addition to a decrease in the glucose uptake and *mTOR* and *EGFR* pathway component expressions, we also detected the expression level of genes responsible for autophagy. Autophagy-inducing gene expression was increased when receiving CAP and SN co-treatment. Autophagy is a regular process that is necessary for the normal functioning of the cell. The transcription factors responsible for autophagy were also evaluated. *ZKSCAN3* and *TFEB* are two important transcriptional factors required for autophagy [34]. The *ZKSCAN3* expression was alleviated 3.5 fold when treated with CAP alone; however, SN treatment non-significantly changed its expression level. The CAP and SN co-treated groups decreased the *ZKSCAN3* level significantly (Figure 5a). Additionally, *TFEB* gene expression revealed an increased expression level while treated with SN only, whilst CAP treatment decreased its expression level (2 times) compared to SN only treatment (7 times) (Figure 5b). The *FOXOs* family (acetylated *FOXO1*) responsible for autophagic induction by binding with *ATG7* and its downregulation is controlled by the autophagy process. Our results suggested that CAP treatment increased the *FOXO1* level 2-fold, while CAP treatment increased it 4-fold. Surprisingly, the CAP and SN co-treatment decreased the expression level up to half of the control, which is an indicator of the progression of autophagy (Figure 5c). The other transcriptional factors are *CRTC2* and *CREBBP*, which also increased while shifting to autophagy. In both transcriptional genes, SN showed a decrease (0.5 times) in the expression level compared to the control. However, CAP treatment increased the *CRTC2* level by 1.5 times and *CREBBP* by 2.2 times, respectively. CAP and SN co-treatment induced its effects and the expression level in this group exceeded 2 times in *CRTC2* and 2.5 times in the *CREBBP* gene, respectively (Figure 5d,e).

We also analyzed the expression status of autophagy-specific genes following exposure by CAP, SN, and CAP + SN in G-361 melanoma cells. *ATG-6/BECLIN-1*, which is one of the key genes, functions as *PI3K* complexes and helps catalyze autophagosomes in the process of autophagy. The SN alone and CAP alone groups increased level from 5 times to 8 times, while the combination of CAP and SN co-treatment significantly increased the *ATG-6* expression level (25 times) compared to the control (Figure 5f).

The protein associated with *ATG-6/BECLIN-1* is known as the activating molecule in *BECLIN 1*-regulated autophagy protein 1 (*AMBRA-1*) and is an important factor at the crossroad between autophagy and apoptosis. The expression level of *AMBRA-1* is directly related to the balance and conversion between autophagy and apoptosis. The CAP alone treatment increased the level of *AMBRA-1* (10 times), while the combination treatment significantly increased its level (31 times), respectively (Figure 5g). The *ATG8/LC3* family is essential for autophagosome biogenesis/ maturation and it also functions as an adaptor protein for selective autophagy. The *MAP1LC3* (microtubule-associated protein 1 light chain 3, hereafter referred to as *LC3*) is a homolog of *ATG8*, which is one of the core proteins present in the conjugation system that helps in elongation and maturation of the autophagosome. The level of *MAP1LC3* is negligible in the control group; however, the CAP and SN group significantly increased the level of the gene by up to 100 times (Figure 5h). Another classical receptor for autophagy is *p62/ SQSTM1*, which is involved in the proteasomal degradation of ubiquitinated proteins. The gene expression level of *SQSTM1* showed a significant increase (~160 times) in the CAP and SN co-treatment group, while it was completely undetectable in the control and CAP only group samples (Figure 5i).

### 2.6. Autophagy Induction in Human G-361 Melanoma Cells

Whether the activation of functional autophagy represents beneficial or destructive strategies for cells under metabolic stress is still questionable [35,36,37].

**Figure 6:**
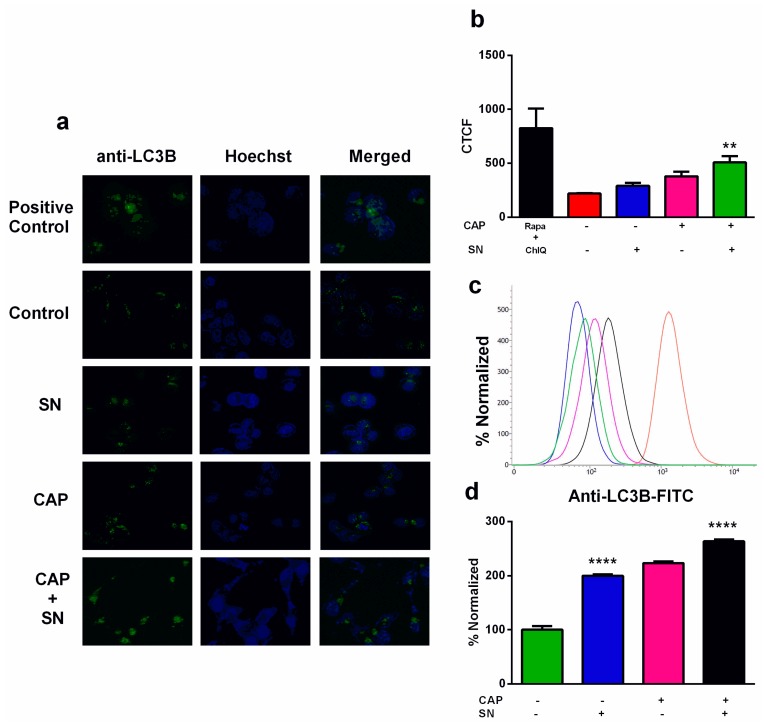
Interpretation of autophagic flux using a kit (Cyto ID® autophagy detection kit) (**a**) by confocal microscopy (green dots represent autophagic sites within G-361 cells and blue stains signify nuclei); (**b**) autophagy image intensities were expressed in terms of CTCF values using bar graphs; (**c**) flow cytometry evaluation of autophagy determination using anti-LC3B FITC-labeled dye; (**d**) FITC expression for autophagy was expressed in terms of a bar graph. Data are the mean ± SE of three experiments. Statistical analysis was performed using a one-way ANOVA test followed by Dunnett’s test for comparisons. Each asterisk represents statistical differences between the treatment and control (** *p* < 0.01 and **** *p* < 0.0001).

One of the classical approaches to inducing autophagy is the inhibition of *mTOR* by rapamycin or other signaling pathways [38,39], which may be regulated by the pH [40]. The autophagy flux was determined by immunocytochemistry (ICC) using a confocal microscope, where we kept the positive control-treated G-361 cells with Rapamycin and Chloroquine. The ICC image showed an increase in image intensity in the positive control group, which confirmed autophagy, and a decrease in the control group (Figure 6a,b). The CTCF values calculated by Image J exhibited maximum fluorescence in the positive control group (825.90), confirming maximum autophagy. The control group revealed a small amount of autophagy (219.57), which was increased in the SN only (291.87) and CAP only (378.94) groups. The autophagic image expression of CAP and SN co-treatment revealed the most fluorescence values (508.39), which confirmed the autophagy process. The autophagy was also investigated using flow cytometry using FITC-labeled anti-LC3B dye which specifically binds autolysosomes (LC3B only) and this revealed the same pattern of an increase in autophagy. The quantitative data from flow cytometry was converted into a histogram (Figure 6c,d) and showed an increase in autophagy when receiving SN only (2 times). The CAP only group showed comparative results that were slightly increased. Additionally, the CAP and SN co-treatment revealed a 2.5 times increase in autophagy compared to the control.

## 3. Discussion

Autophagy is one of the most important mechanisms for the breakdown and reprocessing of long-lived proteins and their aggregates, along with organelles and intracellular pathogens [41]. It helps in maintaining cellular homeostasis in normal conditions by “flushing-out” unwanted/damaged intracellular moieties with the help of lysosomes, providing a potent tool for protection against the accumulation of toxic cellular components [42]. Hence, autophagy is considered as having an important role in various stress stimuli, including several human malignancies and cancer [39,43]. It is recognized that autophagy has a dual role in cancer; the dynamics by which this catabolic process either suppresses or promotes cancer still remain complex and are being debated [35,44]. Efforts to unravel cellular and molecular events that might induce autophagy processes in cancer cells have started to emerge [45,46].

In this article, we have reported that human melanoma G-361 cells treated and incubated with SN (100 nM) for 4 h prior to 180 s CAP treatment showed an increase in cellular stress by a decrease in extracellular pH and intracellular ATP (glucose uptake). The main reason for this is because the decrease in *AKT* phosphorylation due to the CAP and SN co-treated-induced blockage of the *PI3K/AKT* pathway leads to a low pH and reduction in glucose uptake [47], which was evident from a reduction in the 2-NBDG level in G-361 cells [48,49]. A major consequence of abnormal tumor metabolism is the accumulation of metabolic acids within the tumor microenvironment, characterized by a low extracellular pH which is distributed within the tumor mass [50,51]. Figure 2a,b shows that CAP treatment did not alter the extracellular temperature and ranged from 32.46 °C to 33.05 °C (180 s treatment), while the level of extracellular pH decreased as the CAP treatment time increased from 7.46 to 6.87 (180 s treatment). A decrease in extracellular pH helps in the glucose deprivation and de-activation of some crucial cellular pathways and hence cell death [52].

Although autophagy induction mainly occurs in the cytoplasm, it is virtually initiated in the nucleus as part of a transcriptional program controlling lysosome biogenesis/function, mediated by the *MIT/TFE* transcription factors [53]. Our study indicates that the CAP and SN combination dysregulated autophagy-related genes, including *TFEB* and *ZKSCAN3*, which have important functions in connecting *BRAF* signaling to autophagy-lysosome-mediated catabolism in melanoma [54,55]. We have presented evidence for the inactivation of *TFEB* and *ZKSCAN3* by the co-effect of CAP and SN and blockage of the *mTOR/EGFR* pathway that provides a compelling model to explain the suppression of melanoma and resultant autophagy-lysosome activation. In a broader context, the proposed model suggests a novel mechanism by which the loss of signaling through *ZKSCAN, TFEB*, and *FOXO1* results in activation of the autophagy-lysosome pathway in tumor suppression. *ZKSCAN3* is present inside the nucleus and translocates to the cytoplasm in the case of autophagy induction and increases its expression by alleviating its repression of *ATG* genes, which leads to a decreased autophagy process. It activates the transcriptional factor *TFEB*, which later dephosphorylates and translocates back to the nucleus [34]. Hence, the *TFEB* decrease expression level alleviates the autophagy process, which is also evident from our results, where the combination group expressed their levels significantly. Approximately 40–60% of melanoma is due to a mutation in the *BRAF* gene that promotes *MEK-MAPK* and *PI3K-AKT/mTOR* pathway activation and melanoma proliferation [56,57]. Our hypothesis for this study suggested that CAP and SN co-effectively blocked the classical *PI3K/mTOR* and *EGF/MEK* survival pathways, which will eventually lead to the activation of autophagy in G-361 human melanoma. As the results suggested (Figure 4a,b), the gene expression level of *PI3K* in the SN-treated group showed a significant increase, while the CAP alone and combination group exhibited a decreased *PI3K* expression. *mTOR* suppression is a classical pathway inhibition for autophagy induction [58] (Figure 4c) and is also evident from the results. Another pathway (*EGF/MEK*) also showed the significantly highest inhibition in its downstream process (Figure 4a) while receiving CAP and SN in combination. A plausible reason for this is that CAP generates multiple RONS and when it reacts with cell media and reaches cells, it forms many secondary reactive species that react and damage DNA and cytoplasmic *ATM*. This follows the activation of *p53* and *PARP*, which leads to autophagy induction [59].

It was proposed in our previous publication [11] that CAP and SN co-effectively inhibited *BRAF* gene expression in G-361 melanoma cells and hence repressed the mentioned survival pathways which lead to cell death. To address the above mechanism, we also checked the critical role of the other two transcriptional factors *CRTC2* and *CREBBP* [60,61], which were upregulated when receiving CAP and SN and directly related to autophagy induction. To confirm our results, we focused on autophagy-related biomarkers and checked the gene expression in all four treated groups. *AMBRA1* is a main key component of *BECN-1 (ATG-6)* and primarily involved in the formation of *PI3K*-rich membranes during the nucleation phase of autophagy [62,63]. As an initiating regulatory protein of autophagy, *AMBRA1* represents a potential marker of autophagy induction [64]. Our results indicated that treatment with SN and CAP individually expressed the *BECN-1* and *AMBRA-1* expression level, but CAP with SN treatment in combination increased *BECN-1* (25 times) and *AMBRA-1* (22 times) significantly compared to the control sample. Hence, combination treatment of CAP and SN helps in autophagic initiation via the activation of these two genes. *AMBRA-1, LC-3A*, and *SQSTM (p62)* are major biologically distinct markers of autophagic expression [65,66]. The lysosome is a fused and formed autophagosome which results in the transition of *LC3-I* to *LC3-II*, which is a gold standard for autophagy detection [67] and was checked by autophagy in our present work. The gene expression level of *MAP1LC3A*, which also stands for *ATG-8* or *LC3-II*, showed an increase in its expression level while receiving CAP and SN individually, while CAP and SN combination treatment significantly elevated the level (100 times) and clearly indicated the presence of autophagy induction in combination-treated melanoma groups. This can be attributed due to the activation of autophagy transcriptional factors (i.e., *FOXO*), which upregulated the genes *NIX* and *BNIP3* that are directly related to autophagy induction [34]. Additionally, the blocking of survival pathways *AKT* and *ERK* leads to a lower expression level of *mTOR*, which helps in autophagy induction [47].

## 4. Materials and Methods

### 4.1. Chemicals and Antibodies

RPMI-1640, phosphate-buffered saline, and a penicillin-streptomycin antibody cocktail solution were obtained from Welgene, Daegu, Korea. Isoropanol and 4′,6-diamidino-2-phenylindole (DAPI) were purchased from Thermofisher, Seoul, Korea. Diethyl pyrocarbonate (DEPC) was obtained from Biosesang, Gyeonggi, Korea. Trypsin (0.25%) with EDTA and fetal bovine serum was obtained from Hyclone, Seoul, Korea (GE Healthcare Life Sciences, Pittsburgh, PA, USA). The ReverTra Ace® qPCR RT Master Mix c-DNA synthesis kit was purchased from Toyobo, Japan. The SYBR® Green Master mix was procured from Bio-Rad, Seoul, Korea. All the primers for q-PCR studies were obtained from Searchbio, Gyeonggi, Korea. 2-(*N*-(7-Nitrobenz-2-oxa-1,3-diazol-4-yl)amino)-2-deoxyglucose (2-NBDG) was purchased from Biovision, Milpitas, CA, USA and 2′,7′-bis-(2-carboxyethyl)-5-(and-6)-carboxyfluorescein (BCECF-AM) was procured from Santacruz biotechnology, Dallas, TX, USA. The Cyto-ID® autophagy detection kit was purchased from Enzo lifesciences, Farmingdale, NY, USA.

### 4.2. Cell Culture

G-361 human melanoma cells were cultured in RPMI-1640 cell culture media supplemented with 10% fetal bovine serum, 100 U/mL penicillin, and 100 mg/mL streptomycin cocktail and were maintained at 37 °C in a 5% humidified CO_2_ environment. The cells were routinely tested for mycoplasma (MycoAlert™ Mycoplasma Detection Kit, Lonza, Basel, Switzerland). The passage number for G-361 cells was kept constant (P-42) at the time of experiments. The experiments were repeated three times (*n* = 3) using identical conditions.

### 4.3. Characterization of the μ-Dielectric Barrier Discharge (μ-DBD) Plasma System Device

A schematic of the μ-DBD source used in this experiment is shown in Figure 1. The μ-DBD plasma source was fabricated using silver electrodes, which were placed in a coplanar configuration. These electrodes, with a width = 100 µm and thickness = 5 µm, were fabricated above the circular glass substrate (SiO_2_) with a diameter and thickness of 35 mm and 1.8 mm, respectively. The spacing between adjacent silver electrodes on each plane was maintained at 2 mm. In order to prevent the arcing, an additional dielectric layer made up of SiO_2_ (thickness: 50 µm) was coated above the electrodes. Moreover, a hydration prevention layer made up of alumina (thickness: 1 µm) was coated with SiO_2_ to prevent the deposition of water molecules above the dielectric surface. This fabrication process was done by using the technique of photolithography. More details on a similar plasma device can be found elsewhere. One part of the electrode system was connected to the high-voltage power supply and the other part was grounded. In order to operate the discharge for a long time and prevent the heating of electrodes, the discharge was operated in dimming mode [on time (*T*_on_) = 18.77 ms and off time (*T*_off_) = 232.82 ms] by using a DC-AC inverter. The working gas was air [flow rate: 1.5 liters per minute (lpm)] and it was injected from two directions with a cooling fan to reduce the heating. A photograph of the μ-DBD discharge operated by air is shown in Figure 1a, b. This plasma source was used to treat G-361 cells for 180 s.

### 4.4. Estimation of Extracellular pH and Temperature and Number of Melanoma Cells

RPMI-1640 serum-free media were placed in 48-well plates (1 mL: per well) and exposed to μ-DBD plasma using air as the feeder gas for 30, 60, 180, and 300 s. After exposure, the temperature and pH of the media were measured immediately in triplicate with an infrared (IR) camera (Fluke Ti100 Series Thermal Imaging Cameras, Everett, WA, USA) and pH meter (Eutech Instruments, Singapore). The G-361 cells were counted and DIC images (Leica, DMi8, Wetzlar, Germany) were taken after different CAP exposure times.

### 4.5. Glucose Uptake

G-361 cells after culture were treated with SN (100 nM), CAP (180 s), and a combination of SN + CAP and kept for the next 24 h in a CO_2_ incubator. After incubation, G-361 cells were incubated with 100 μM 2-NBDG, which is a fluorescent derivative of 2-deoxy-d-glucose, for 45 min. The cells were trypsinized, collected, and washed with PBS and then analyzed by flow cytometry to collect green fluorescence. In a separate experiment, G-361 cells were fixed and permeabilized separately using 10% ethanol and 3.7% paraformaldehyde. They were then incubated with DAPI for 10 min to counterstain the nucleus, along with 2-NBDG. The fluorescence staining intensity and intercellular locations were examined using an Olympus IX83-FP confocal microscope (Tokyo, Japan).

### 4.6. qPCR Gene Analysis (Autophagy Related and Transcriptional Factor)

Any alterations in the cellular level are associated with changes in the gene expression at the mRNA level. To clarify this at molecular levels, we identified genes responsible for the induction of *mTOR*-mediated autophagy and related transcriptional factors and checked their levels by the production of plasma-generated reactive species. The desired primers for *mTOR* and *RAS/MEK*-mediated autophagy and its relevant transcriptional factors were designed and synthesized. G-361 melanoma cells were grown in Petri plates with a size of 35mm^2^ and treated with CAP, SN, and CAP + SN, respectively, as described before. After incubation for 24 h, the cells were trypsinized and collected for RNA isolation using Trizol (Invitrogen, Carlsbad, CA, USA), after which q-PCR was performed with a Biorad 2X SYBR green mix (Biorad, Seoul, Korea). Reactions were carried out in a Biorad thermal cycler (Biorad, Seoul, Korea), and the results were expressed as the fold change calculated with the 2^−ΔΔCt^ method relative to a control sample. Meanwhile, *18S r-RNA* was used as an internal normalization control. All primers were purchased from Searchbio, Gyeonggi, Korea. Quantitative real-time PCR was performed according to the forward and reverse primer sequences listed in Supplementary Tables S1 and S2. 

### 4.7. Evaluation of Autophagy by Immunocytochemistry (ICC), Flow Cytometry, and ELISA

G-361 melanoma cells were used to monitor autophagy fluorescence (a cyto-ID® autophagy detection kit specifically measures autophagic vacuoles and autophagic flux). To get insight into the functional role of autophagy in the mentioned groups, we analyzed autophagy in human melanoma using the Cyto ID autophagy detection kit, which specifically tags the formation of autophagosomes (LC3B formation). This kit contains the anti-LC3B (anti LC-3II) antibody which stains autophagic sites and CTCF values were determined using image-J software. The melanoma cells were plated in 35 mm^2^ Petri dishes and treated with CAP, SN, and CAP + SN for 24 h, respectively, as per the standard experimental procedures mentioned above. Rapamycin (500 nM) and chloroquine (10 μM) were added to positive control samples and acted as autophagy inducers. After 18 h with autophagy inducers and 24 h with CAP, SN, and CAP + SN, the media was replaced and washed with 1× assay buffer (in 5% FBS), which was further kept in 1 mL 1× assay buffer (without FBS). The cells were stained with Cyto-ID® green detection reagent and counterstained with Hoechst 33342. The autophagy was determined by using an Olympus IX83-FP confocal microscope (Tokyo, Japan) and flow cytometry was performed by BD FACSVerse using the FACS suite software (Becton Dickinson and Co., Franklin Lakes, NJ, USA).

### 4.8. Statistical Analysis

All of the data are expressed as the mean ± SE of triplicates. The statistical software GraphPad Prism 6.0 (Microsoft Corporation, Redmond, WA, USA) was used for statistical analysis via one-way analysis of variance (ANOVA) followed by Dunnett’s test.

## 5. Conclusions

Our present study delivers further support to therapeutic strategies targeting autophagy to kill cancer cells. This article suggests a plausible method of killing human melanoma cells using the same conditions and shows that autophagy plays a vital role in the eradication of melanoma by using CAP and SN. G-361 melanoma cell reduction was produced because of a decrease in the extracellular pH, which results in a reduction in the intracellular glucose level with the inhibition of *mTOR* and *EGF* survival pathways and leads to autophagy activation. However, more studies are needed to dissect the mechanisms that regulate autophagy in aggressive and different melanoma cells for the development of therapeutic strategies that will benefit patient outcomes.

## Supplementary Materials

Supplementary materials can be found at https://www.mdpi.com/1422-0067/21/6/1939/s1.

## Abbreviations

CAP	Cold Atmospheric Plasma
SN	Silymarin Nanoemulsion
RONS	Reactive Oxygen and Nitrogen Species
*PI3K*	Phosphatidyl Inositol 3 Kinase
*mTOR*	Mammalian Target of Rapamycin
*MEK*	Mitogen-Activated Protein Kinase
ATP	Adenosine Triphosphate
*ZKSCAN3*	Zinc Finger with KRAB And SCAN Domains 3
*TFEB*	Transcription Factor EB
*FOXO1*	Forkhead Box Protein O1
*CRTC2*	cAMP-Response Element Binding Protein Regulated Transcription Coactivator 2
*CREBBP*	cAMP-Response Element Binding Protein
*BECN-1*	Beclin 1
*AMBRA-1*	Autophagy and Beclin 1 Regulator 1
*MAP1LC3A*	Microtubule Associated Protein 1 Light Chain 3 Alpha
*SQSTM*	Sequestosome-1
*EGFR*	Epidermal Growth Factor Receptor
