# Supplementary material for: Cold Atmospheric Plasma and Silymarin Nanoemulsion Activate Autophagy in Human Melanoma Cells
Source: Int J Mol Sci. 2020 Mar 12;21(6):1939. doi: 10.3390/ijms21061939 (PMC7139470; doi:10.3390/ijms21061939)
Supplement: Supplementary file 1 [file ijms-21-01939-s001.pdf]

International Journal of
Molecular Sciences

MDPI

Cold atmospheric plasma and silymarin nanoemulsion activate Autophagy in human melanoma cells

Supplementary Table 1

S1: List of Primers related to *PI3K-mTOR* and *EGFR* pathways

Name	Sequence (5′ – 3′)
PI3K F	GATAGTTGTCTCCCCTCCTTCTC
PI3K R	GTTTGGTTCTTTGCCTCATTTC
mTOR F	GCGTCCCTACCTTCTTCTTC
mTOR R	TCAAATCCCTTCTCTGCTTCT
EGFR F	GCTATGAGATGGAGGAAGACG
EGFR R	GAGGAGGAGTATGTGTGAAGGA
HRAS F	AAGTGTGTGCTCTCCTGACG
HRAS R	GTCCTTCCTTCCTCCTCCTT
MEK F	CTCACCATCAACCCTACCATC
MEK R	TCTTCTGCTGCTCGTCAAGT

S2: List of Primers responsible for autophagy related transcriptional factor and its specific genes

Name	Sequence (5′ – 3′)
18S RNA F	CAGGTCTGTGATGCCCTTAGA
18S RNA R	GCTTATGACCCGCACTTACTG
ZKSCAN3 F	TGATAGGAGGCTACGGGAGAG
ZKSCAN3 R	AATCTGACCAACTGTGGAGGA
TFEB F	ATCAATACCCCCGTCCACTT
TFEB R	ACATCATCCAACTCCCTCTCA
FOXO1 F	GTTGGGCAGGAAAGTGATGT
FOXO1 R	GCAGAAGGGAGAATGAGATGAA
CRTC2 F	CACCTCATCACCATCCCTCT
CRTC2 R	AACCCCTTGCTTTTTCTCATTC
CREBBP F	GCATCACCTTTTCCCTTTCA
CREBBP R	AACATCAATCCACCCTTCCA
BECN-1 F	AGGAGAGGAGCCATTTATTGA
BECN-1 R	CCACTATCTTGCGGTTCTTTT
AMBRA1 F	TGACCAATAACAACCACCTTCT
AMBRA1 R	ATCTTCCTCTCCACCCTGAC
MAPLC3 F	TACGAGCAGGAGAAAGACGAG
MAPLC3 R	GCAGAGTAGGTGGGTTGGTG
SQSTM1 F	CAGCACAGAGGAGAAGAGCA
SQSTM1 R	AGGAAACATCAGCACACACAC
